# The role of oxidative post-translational modifications in type 1 diabetes pathogenesis

**DOI:** 10.3389/fimmu.2025.1537405

**Published:** 2025-02-14

**Authors:** Ghadeer Alhamar, Chiara Vinci, Valentina Franzese, Flavia Tramontana, Nelig Le Goux, Johnny Ludvigsson, Ahuva Nissim, Rocky Strollo

**Affiliations:** ^1^ Department of Immunology and Microbiology, Dasman Diabetes Institute, Dasman, Kuwait; ^2^ Center for Diabetes Research, Université Libre de Bruxelles, Brussels, Belgium; ^3^ Department for the Promotion of Human Science and Quality of Life, San Raffaele Open University, Rome, Italy; ^4^ Department of Medicine, Fondazione Policlinico Universitario Campus Bio-Medico di Roma, Rome, Italy; ^5^ Department of Medicine, Università Campus Bio-Medico di Roma, Rome, Italy; ^6^ Biochemical Pharmacology, William Harvey Research Institute, Queen Mary University of London, London, United Kingdom; ^7^ Crown Princess Victoria Children’s Hospital and Division of Pediatrics, Department of Biomedical and Clinical Sciences, Linköping University, Linköping, Sweden

**Keywords:** post-translational modifications, type 1 diabetes, oxidative stress, neoepitopes, autoimmunity, insulin, autoantibodies, t cell

## Abstract

The pathogenesis of type 1 diabetes (T1D) involves a complex interplay of genetic predisposition, immune processes, and environmental factors, leading to the selective destruction of pancreatic beta-cells by the immune system. Emerging evidence suggests that intrinsic beta-cell factors, including oxidative stress and post-translational modifications (PTM) of beta-cell antigens, may also contribute to their immunogenicity, shedding new light on the multifaceted pathogenesis of T1D. Over the past 30 years, neoepitopes generated by PTMs have been hypothesized to play a role in T1D pathogenesis, but their involvement has only been systematically investigated in recent years. In this review, we explored the interplay between oxidative PTMs, neoepitopes, and T1D, highlighting oxidative stress as a pivotal factor in immune system dysfunction, beta-cell vulnerability, and disease onset.

## 1 Type 1 diabetes: a disease of the immune system or of the beta-cell?

Type 1 diabetes (T1D) is a chronic autoimmune disease characterized by the destruction of insulin-producing pancreatic beta-cells, whereas neighboring alpha, delta, and epsilon cells are mainly spared (1). This targeted immune response leads to progressive loss of endogenous insulin secretion, resulting in lifelong dependence on exogenous insulin therapy. Decades of research have highlighted the critical role of genetic predisposition, particularly the inheritance of high-risk HLA class II alleles, in shaping susceptibility to T1D (2, 3). These HLA molecules influence the presentation of beta-cell-derived autoantigens to autoreactive T cells, a pivotal event in disease onset (4). Beyond the HLA region, non-HLA genes, such as *INS*, *PTPN22*, *CTLA4*, and *IL2RA*, have been implicated in modulating immune tolerance and T cell activation (5). Together, these genetic factors converge to foster an autoimmune *milieu* characterized by the generation of autoantibodies and dysregulated T cell responses. While these findings underscore the central role of autoreactive T cells, emerging evidence suggests that intrinsic beta-cell factors, including oxidative stress and post-translational modifications (PTM), may also contribute to their immunogenicity, shedding new light on the multifaceted pathogenesis of T1D (6).

Building upon the understanding of genetic and immune factors in T1D, the detection of autoantibodies against key beta-cell antigens—such as insulin, glutamate decarboxylase (65) (GAD65), zinc transporter 8 (ZnT8), and insulinoma antigen-2 (IA-2)—provides a powerful diagnostic tool (7). These autoantibodies are critical markers of autoimmunity and enable the stratification of T1D into three progressive stages (8). In Stage 1, the presence of multiple autoantibodies indicates a breach in immune tolerance, even in the absence of dysglycemia. Stage 2 is characterized by glucose intolerance, signaling the onset of beta-cell dysfunction. Stage 3 corresponds to clinically manifest diabetes, defined by hyperglycemia or elevated HbA1c. The combined detection of these autoantibodies serves as the strongest predictor of disease progression, yet their precise role in beta-cell destruction remains enigmatic.

Hence, the questions arise: why has the immune system specifically turned on the beta-cells? Is the immune attack coming from factors outside the cell, or is it initiated by factors within the cell meaning: is the beta-cell responsible for its own demise? These provocative questions were posed at the EASD congress in 1985 and later expanded upon in a seminal article in 1986 by Gian Franco Bottazzo (9), who attempted to understand whether the destruction of the beta-cell was a result of self-destruction (“suicide”) or immune attack (“homicide”) (10).

Recent research has revisited this hypothesis, exploring the possibility that beta-cells play an active role in their own destruction. Oxidative stress, along with PTM of beta-cell proteins, has emerged as a potential mechanism by which the beta-cell becomes immunogenic, effectively marking itself as “non-self” and triggering an immune response. These findings suggest that T1D pathogenesis may involve a complex interplay between external immune attacks and intrinsic beta-cell vulnerabilities.

## 2 Oxidative stress in type 1 diabetes

Inflammation and oxidative stress are key players in the development of autoimmune disorders, resulting in an overload of reactive oxygen species (ROS) (11), with T1D being no exception. Two main mechanisms are potentially involved in the increase of ROS within the islet microenvironment: 1) reactive oxidants could be released by leukocytes during insulitis; 2) altered redox state within beta-cell under stress could facilitate reactive oxidants production. In the early stages of T1D, the pancreas is infiltrated by immune cells targeting the islets and their insulin producing beta-cells. This creates a pro-inflammatory environment accelerating the disease process. At a later stage, dysglycemia develops, which can further create a vicious cycle amplifying oxidative stress and generation of ROS.

During typical mitochondrial respiration, the result is ATP and free radicals. These free radicals are highly reactive and have high potential to impact, modify, or destroy other molecules (12). Hydrogen peroxide (H_2_O_2_) and lipid peroxides, superoxide and the hydroxyl radical (•OH) are the main forms of metabolically derived ROS; peroxynitrite and nitric oxide are the major forms of reactive nitrogen species (RNS) (12). H_2_O_2_ may act as substrate in the Fenton reaction that leads to the formation of •OH in the presence of divalent metals, such as iron or copper. •OH radicals have a half-life of 10^-9^ sec and react instantaneously with biological molecules by subtracting a hydrogen atom. H_2_O_2_ may also interact with chloride ions present in plasma leading to the formation of hypochlorite (HOCl). HOCl is a potent antimicrobial agent generated by the enzyme myeloperoxidase (MPO) and released by polymorphonuclear leukocytes (PMN) during phagocytic degranulation. In contrast with •OH, HOCl is more stable with a half-life of hours or days (13–15). During inflammation, the vascular endothelium and inflammatory cells may produce nitric oxide radical (NO^•^) via the nitric oxide synthase (NOS); NO^•^ may react with superoxide to generate the unstable molecule ONOO^-^ which have a half-life of 0.05-1 sec (16). When kept at physiological levels, these ROS and RNS play crucial roles in normal biological development, including cellular signaling, synthesis of hormones and phagocytosis (17). However, when there is an imbalance in the production and removal of ROS, this leads to oxidative stress and potential pathology (18).

### 2.1 Susceptibility to oxidative stress and beta-cell fragility

Several antioxidant systems are in place to maintain homeostasis. These include antioxidant enzymes, low molecular mass antioxidants such as GSH, uric acid and vitamins B, C and E, and sequestration and repairing systems. The most important intracellular antioxidant enzymes are glutathione peroxidase (GPX), catalase, and superoxide dismutase (SOD) (12). Interestingly, although pancreatic islets are highly metabolically active and play a critical role in maintaining the body’s glucose homeostasis, research has shown that the pancreatic beta-cells possess comparatively low levels of antioxidants (19).

When compared to liver antioxidant levels, pancreatic islets contain 1% catalase, 29% SOD1, and 2% GPX1 activity, leaving the beta-cells with considerably low antioxidant protection and vulnerable to oxidative stress. Flekac et al. demonstrated significant difference in the distribution of *Sod1* and *Sod2* alleles and genes among individuals with T1D and type 2 diabetes and non-diabetic controls, but not in *CAT* gene, which encodes for catalase (20). This was linked to reduced serum SOD activity in patients with diabetes compared to the control subjects.

The influx of free oxidative radicals coupled with an insufficient antioxidant response, gives rise to the perfect environment for oxidative stress and the oxidation of proteins, nucleic acids, and lipids. An abundance of free radicals within the cellular environment can also trigger intracellular stress-sensitive pathways, causing further stress and damage to the cells. These include signaling pathways such as p38 mitogen-activated protein kinases (p38 MAPK), nuclear factor kappa B (NF- κB), protein kinase C (PKC), and Advanced glycation end product/receptor for AGE (AGE/RAGE), pathways to name a few (21, 22).

The function of the pancreatic beta-cell can also be challenged by certain genetic variants in the gene encoding insulin (*INS*). As reviewed by Roep et al., protective variants of *INS* resulted in increased *INS* expression in the thymus, therefore increasing the probability that the immune system will be educated to avoid immune reactivity to insulin. However, differences in *INS* activity in pancreatic islets have also been linked to these genetic polymorphisms, as well as effects on beta-cell function and resilience (6).

Eizirik et al. studied other candidate genes for T1D, acting both at the immune system and at the pancreatic beta-cell level. They found that 61% of the candidate genes for T1D are consistently expressed in human pancreatic islets. Furthermore, the expression of many of these genes changed following exposure to pro-inflammatory cytokines or dsRNA (a by-product of virus infection), agents that may contribute to triggering oxidative stress and T1D. Particularly, many key beta-cell functions were modified by cytokines. Most important responses were those related to inflammation, innate immune response and apoptosis. Notably, cytokines induce significant upregulation of genes involved in IFN-γ signaling, NF-κB regulation, antigen presentation, and free radical scavenging, further linking oxidative stress to beta-cell fragility and T1D pathogenesis (23).

### 2.2 Experimental and epidemiological evidence linking oxidative stress to type 1 diabetes

There is substantial evidence supporting a pathogenic role for oxidative stress in T1D. Several of the main putative etiopathogenic factors linked to T1D have been shown to be or are potentially able to generate oxidative stress (24–26). For example, it is generally known that numerous virus infections, including those suggested to be involved in T1D, exert many kinds of oxidative stress in the host. Superoxide production following Coxsackieviruses B3 infection may exacerbate pancreatic beta-cell destruction in an experimental model of T1D by influencing proinflammatory macrophage responses (27), thus mechanistically linking oxidative stress, inflammation, and diabetogenic virus infections. Secondly, circulating markers of oxidative stress are increased not only in patients with established T1D, but also in euglycemic subjects at risk for T1D (28, 29). Interestingly, studies have indicated that first-degree family members of patients with T1D also show significantly lower levels of serum antioxidants. These findings suggest a familial precedence for the dysregulation of ROS and antioxidants seen in T1D (28). A longitudinal study including patients recruited into the Diabetes Autoimmune Study in the Young (DAISY) cohort assessed children at-risk of T1D from birth up to 14 years. This study found irregular expression of 16 proteins involved in oxidative stress in these patients even before the initiation of seroconversion to autoantibody positivity (30). This suggests that increased oxidative stress and ROS may play a bigger role in the development of T1D than previously thought. Oxidative stress exists in the early stage of T1D progression, prior to earliest sign of islet-autoimmunity (30). Thirdly, cohort studies have shown that iron overload is associated with increased risk of T1D (31, 32). Iron salts act as catalyst in Fenton chemistry by converting hydrogen peroxide to ^•^OH radicals that are highly oxidizing. Finally, diabetes can be induced experimentally in rats by feeding with alloxan or streptozotocin (STZ); these two substances work by generating ROS and selective damage to beta-cells (33). Of note, low dose STZ leads to insulitis and to an immunological alteration in the islets that has been hypothesized to be the result of a neoantigenic epitope elicited by STZ toxicity (34).

## 3 Post-translational modifications

In normal physiological conditions, proteins are subjected to PTMs, expanding on the functionality and diversity of proteins (35). PTMs can impact the function, dynamics and structure of affected proteins. PTMs are generally characterized as either reversible or irreversible modifications (36), and enzymatic or non-enzymatic (37). In many cases, PTMs are essential for the final structure of the proteins and their biological activity, such as gene expression and regulation, DNA repair, cell cycle control and signal transduction (36). For instance, enzymatic reversible PTMs include phosphorylation, which is an essential process in the activation of protein channels and enzymatic activity (38). Another example includes the methylation of histones within the nucleus, which behaves as *gene silencers*, playing an important role in gene regulation catalyzed via histone methyltransferases, and removal of methyl group catalyzed by histone demethylases (39).

Typically, cells are responsible for maintaining their own homeostasis and cellular viability. However, in cases of excess and persistent inflammation, oxidative stress, and abnormal glucose levels, cellular proteins are at risk of developing non-enzymatic PTMs. These PTMs may alter protein function, dysregulating homeostasis and causing irregularities in protein 3-dimensional conformation (37). These include, but are not limited to, protein oxidation, deamidation, glycation, and isomerization (40). Several reports have documented amino acid side chains being irreversibly modified by ROS, these include lysine, cysteine, arginine, and histidine to name a few (41). Interestingly, the progression of several pathologies has been linked to irreversible non-enzymatic protein modification. For instance, the reactivity of proteins with 4-hydroxy-2-nonenal and malondialdehyde have been associated with the progression of Alzheimer’s disease (41). The oxidation of amino acid side chains histidine, cysteine and tyrosine have been associated with cystic fibrosis while carbamylation of cysteine residues are implicated in cardiomyopathies and atherosclerosis (42, 43), and glycation and carbonylation of amino acids including lysine and cysteine have been associated with diabetes.

**Figure 1:**
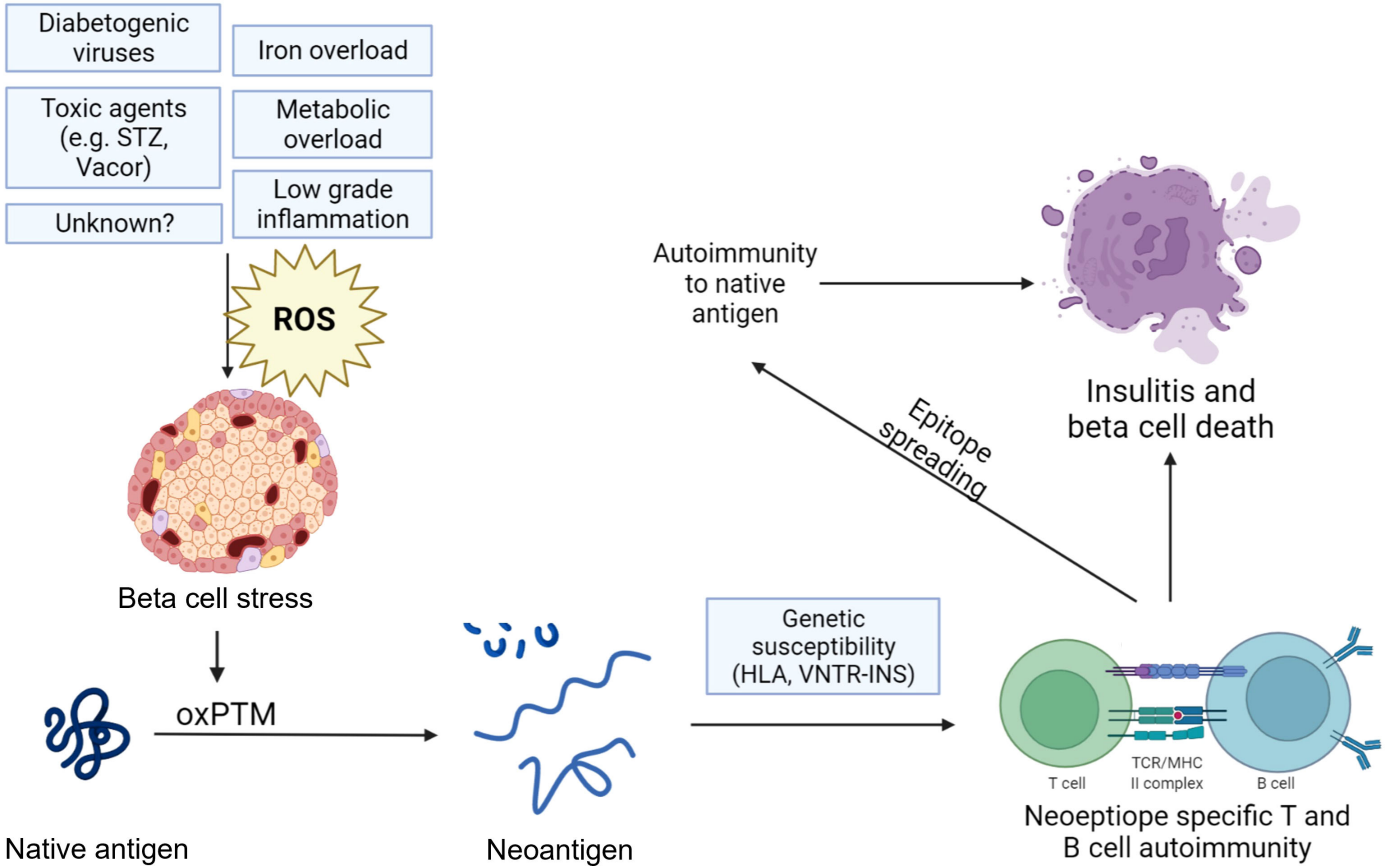
Hypothesized mechanism of neoepitope generation by oxidative post-translational modifications of beta-cell antigens in type 1 diabetes. Various factors such as viral infections, toxins, and metabolic overload can induce oxidative stress and a state of beta-cell stress, leading to oxidative post-translational modifications (oxPTMs) and, subsequently, neoepitopes that may trigger autoimmune responses. These responses can amplify reactions against native antigens or involve them through epitope spreading. The beta-cell becomes a victim of external events that lead to an accumulation of oxPTM, transforming it and unfairly marking it as a target for the immune system. The figure was created with the help of bioRender.com.

Research has begun to investigate the role of oxidative PTMs (oxPTMs) in inciting the immune response in autoimmune conditions. oxPTMs and other PTMs are suggested to induce neoepitopes or new antigens that are less inclined to be presented during positive and negative selection in the thymus and are likely to be treated as foreign epitopes (11, 44, 45). This has been attributed to oxPTMs and the subsequent neoepitopes influencing several immunological phenomena, including epitope spreading, molecular mimicry, antigen coupling, and exposing of cryptic epitopes, with these mechanisms influencing the loss in immune tolerance. Neoepitopes are believed to behave as pathogen- or danger-associated molecular patterns (PAMPS/DAMPS), which the immune system can detect through pattern recognition receptors (PRRs). These PRRs include Toll-like receptor 4 (TLR4), scavenger receptors, receptor of advanced glycation end products (RAGE), and natural IgM antibodies. Increased and continuous exposure of neoepitopes may cause increased exposure of autoantigens to the immune system, a mechanism that is known to inducing class switching and the adaptive immune response (**Figure 1**) (11).

### 3.1 Chemistry of oxidative post-translational modifications

Protein oxidation occurs due to the reaction of ROS or RNS with amino acid residues of proteins, with these reactions being either reversible or irreversible in nature (**Table 1**) (46). The induced protein modifications by elevated ROS levels can occur via 60 different pathways, including glycation, oxidation and carbonylation (11, 47–49). Four major mechanisms are currently attributed to oxPTMs: 1) oxidative cleavage with or without amino acid modification; 2) amino acid modification; 3) generation of intra-molecular and inter-molecular crosslinks; 4) protein conformational changes. There is a possibility that a combination of all four mechanisms is responsible for the formation of neoantigens. For instance, evidence suggests that proteins may crosslink through covalent disulfide bonds or formation of dityrosine when exposed to oxidative stress and ROS (50). Another common mechanisms to induce protein oxidation involves metal catalyzed oxidation (MCO), notably when introducing carbonyl groups to susceptible proteins (51).

**Table 1:** Oxidative post-translational modifications induced by different reactive oxidants and main amino acids targeted.

Modification	Target Amino Acid	Modified Amino Acid/Byproduct	Mechanism
Non-enzymatic glycation	Lysine	CML Pentosidine AGEs	Reaction with reducing sugars under oxidative stress
	Arginine	Pentosidine AGEs	
	Phenylalanine	AGEs	
Oxidation	Methionine	Methionine sulfoxide Methionine sulfone	Oxidation by hydroxyl radicals (•OH) or peroxides
	Cysteine	Cysteine sulfenic acid	
	Tyrosine	DOPA	
	Phenylalanine	Tyrosine	
	Tryptophan	Hydroxytryptophan, Kynurenine	
	Histidine	2-Oxohistidine, Aspartate Glutamate	
	Proline	Hydroxyproline Glutamate	
Chlorination	Tyrosine	3-Chlorotyrosine, Dichlorotyrosine	Reaction with hypochlorous acid (HOCl)
Nitrosylation	Cysteine	S-nitrosocysteine	Reaction with nitric oxide derivatives (NO•, ONOO-)
	Tyrosine	3-Nitrotyrosine	
	Tryptophan	6-Nitrotryptophan	
	Lysine	Nitrosylated lysine	
Carbonylation	Lysine	Α-Aminoadipic semialdehyde	Irreversible reaction where a Carbonyl group (C=O) is added to the amino acid side chain
	Arginine	Glutamic semialdehyde	
	Proline	Glutamic semialdehyde	
	Threonine	Α-Amino-B-ketobutyric acid	

AGEs, Advanced glycation end-products; CML, Carboxymethyl-lysine; DOPA, DihydroxyPhenylalanine.

Regarding the oxidation of cysteine residues, it requires a pKa lower than physiological pH (7.4). This low pKa will allow the cysteine -SH groups to be thiolated, it is these thiolated residues that are redox reactive (46). The oxidation of cysteine residues begins with the production of sulfenic acid, which leads to the formation of other oxidative products. These include the formation of intra- and inter-protein disulfides, *S*-sulfenylation, *S*-sulfinylation, and *S*-sulfonylation (52). These oxidative modifications have been highlighted because they are able to go on and induce further oxidative stress and subsequent protein modifications (53). This review will focus on the role of glycation, oxidation and protein carbonylation.

#### 3.1.1 Glycation

Glycation is a non-enzymatic spontaneous chemical reaction between a protein and a reducing sugar. This process is accelerated by ROS, which can swiftly cleave glucose, leading to molecular rearrangements, radical formation, and the production of dicarbonyl compounds. These highly reactive products interact readily with free amino groups on protein residues, ultimately promoting glycation (54, 55). Typically, glucose is the major glycating agent within biological systems. Glucose has the capability to react with the N-terminal amino group of lysine residues, forming a *Schiff’s base*, which can undergo rearrangements to yield fructosamines (N-(1-deoxy-D-fructos-1-yl). In later stages, fructosamines begin to degrade and form more stable advanced glycation end products (AGEs) (56). There are several amino acids more susceptible to glycation, namely lysine and arginine, especially in cases of increased oxidative stress (57). Research has shown several other glycating agents that are of concern, these include methylglyoxal (MG), 3-deoxyglucosone, and glyoxal. Minor glycating agents to note are Nϵ-carboxyethyl-lysine derived from MG, and Nϵ-carboxymethyl-lysine (CML) and Nω-carboxymethyl-arginine (CMA) derived from glyoxal (56).

Glycation has been extensively studied within health and disease, specifically within the context of diabetes, aging, diabetic complications, and arthritis. Glycated proteins have already been used in monitoring and managing the progression of diabetes, which includes glycated hemoglobin (HbA1c), serum fructosamine, and glycated albumin (56). HbA1c arises from the spontaneous reaction of glucose with valine residues of the N-terminus of hemoglobin beta chains (58).

#### 3.1.2 Oxidation

Elevated ROS levels have been previously associated with chronic disease, such as cancer, neurological diseases and diabetes (18, 59–61). One of the most reactive and potent reactive oxidants is the •OH, which can oxidize proteins, DNA, and other biological compounds (62–64). The •OH is able to modify amino acids, peptides and proteins via several reactions, including hydrogen subtraction, addition, and electron transfer. Typically, the •OH targets amino acid sidechains or protein backbones (**Table 1**) (65).

The primary reaction involved in the cleavage of protein backbones begins with H abstraction at the α-carbon position, closely followed by reaction with O_2_, producing the peroxyl radical. Collectively, this eventually leads to the cleavage and fragmentation of the protein backbone, yielding carbonyl and amide fragments (66). In terms of reactivity with H_2_O_2_, histidine, tyrosine, phenylalanine and arginine are capable of preferentially taking oxygen, whereas methionine competitively takes up oxygen (67). Oxidative PTMs have been seen in several autoimmune diseases, including celiac disease, rheumatoid arthritis, and irritable bowel syndrome (68, 69).

#### 3.1.3 Carbonylation

One of the major products of protein oxidation is the formation of carbonylated proteins (51, 70). Protein carbonylation can occur via secondary reactions with reducing sugars, their oxidation products or with 4-hydroxynonenal, a lipid peroxidation product (71).

Protein carbonylation is an irreversible, metal catalyzed oxPTM of the side chains of amino acids, mainly lysine, proline, arginine, and threonine (72, 73). Carbonyls can interact and modify the α-amino groups of lysine residues, forming potential inter- and intra-molecular crosslinks. These crosslinks can induce protein aggregation, making the proteins difficult to degrade and hindering normal proteolytic pathways (51). This modification inflicts many harmful and dangerous effects on multiple intracellular enzymatic mechanisms, with mitochondria being particularly susceptible to carbonylation.

The irreversible nature of carbonyl modifications makes them ideal targets for immune recognition in an autoimmune context. The rate of carbonylation increases with either cytokine stress or elevated oxidative stress within beta-cells. While implicated in autoimmune responses in T1D, studies have also suggested that carbonylated proteins may serve as markers of beta-cell dysfunction and oxidative damage in type 2 diabetes (74).

### 3.2 Synergies between oxidative stress and other PTM pathways

Oxidative stress may also interact with enzymatic PTM pathways, amplifying their effects (75). ROS can influence the modification of specific protein motifs, alter metabolic pathways, and induce secondary effects through cellular processes such as apoptosis, inflammatory responses, and NETosis (75). These interactions contribute to autoimmune responses and tissue pathology by increasing protein immunogenicity, altering solubility, and impairing protein clearance.

An example is the interaction between oxidative stress and citrullination, an enzymatic PTM catalyzed by peptidylarginine deiminases (PADs), which convert arginine residues into citrulline. ROS have been shown to promote citrullination of histone H3 in granulocytes via PAD4, a process critical for NETosis, an inflammatory mechanism implicated in autoimmunity (76, 77). Exogenous H_2_O_2_ can also induce histone citrullination and NET formation in neutrophils, with the extent and timing of ROS production being crucial (77, 78). Interestingly, supraphysiological ROS levels may inhibit PAD activity, as observed in studies showing that H_2_O_2_ concentrations above 40 µM directly suppress PAD2 and PAD4 activity (78). These findings suggest a complex interplay between intracellular and extracellular ROS concentrations in modulating PAD activation and citrullination, with additional influences from the redox balance.

Oxidative stress may also indirectly contribute to deamidation through its impact on cellular processes. Inflammatory cytokines, which are known to activate ER stress pathways, have been shown to induce both citrullination and deamidation of beta-cell proteins (79, 80), such as GRP78 and proinsulin. This link highlights the role of cytokine-induced oxidative and ER stress in shaping the PTM landscape, potentially exacerbating beta-cell vulnerability and immune recognition (81, 82).

## 4 Oxidative PTM and neoepitopes associated with type 1 diabetes

### 4.1 Insulin

Within recent years, PTMs have been implicated in the production and secretion of insulin. For instance, acetylation, O-GlcNAcylation, and ubiquitination have been shown to be involved in insulin gene transcription, and phosphorylation has a role in mediating the signaling cascades needed for insulin secretion (83). However, current research has suggested that modified antigens play a critical role in the autoimmunity characteristic of T1D, with insulin potentially being the predominant autoantigen (84).

In 2005, Mannering et al. demonstrated that harvested CD4^+^ T cell clones directed toward proinsulin isolated from a patient with T1D, were able to recognize the first 13 amino acids of human insulin A-chain. T cell recognition was dependent on the formation of disulfide bonds between cysteines A6 and A7 (**Table 2**). This study was one of the first to suggest that T cell recognition of insulin could rely on natural PTM, such as disulfide bond formation, rendering the molecule immunogenic and potentially triggering an autoimmune response (85).

**Table 2:** Immune responses to oxidative post-translational modifications identified in type 1 diabetes.

Autoantigen	Modification	Target residues	Type of immune response	Peptide sequence	Reference
*Insulin, chain A*	Oxidation (Disulfide bond)	6C and 7C	CD4^+^ T cell	epitope A:1–13, GIVEQ**CC**TSICSL	(85)
*Insulin, chain B*	Oxidation (•OH and HOCl)	24F, 5H, 16Y, 26Y, 25F, 14Y, 10H, 17L, 19C	Autoantibodies, CD4^+^ and CD8^+^ T cell, CD4^+^ T cell	epitope B:11-30, LVEAL**YL**V**C**GERG**FFY**TPKT epitope B:21-30, ERG**FFY**TPKT epitope B:21-31, ERG**FFY**TPKTR	(86, 87)
	Oxidation (in silico)	24F, 25F	HLA Class I	epitope B:21-30, ERG**FF**YTPKT	(88)
	Oxidation (Cu in ambient air)	19C	γδ T cell	epitope B:9-23, SHLVEALYLV**C**GERG	(89)
	Chlorination (HOCl)	26Y, 16Y	Autoantibodies		(86)
*GAD65*	Oxidation (•OH)	Unknown	Autoantibodies, neoepitope unknown		(90, 91)
*Type II Collagen*	Oxidation (•OH, HOCl), Glycation, Chlorination (HOCl), Nitrosylation (ONOO-)	Unknown	Autoantibodies associated to *HLA*-DRB1*04 and rheumatoid arthritis, neoepitope unknown		(92)
*Prolyl-4-hydroxylase beta*	Carbonylation	Unknown	Autoantibodies, neoepitope unknown		(93)

Residues targeted by oxPTM are highlighted in bold within the native peptide sequence.

Our group has found that auto-reactivity to insulin might also be directed towards neoepitopes induced by ROS. In our study, a higher prevalence of auto-reactivity to oxPTM-insulin (oxPTM-INS) was observed in individuals with new-onset T1D compared to native insulin. Autoantibodies against oxPTM-insulin (oxPTM-INS-Ab), measured by ELISA, demonstrated highly sensitive for T1D diagnosis in this cohort, detecting over one third of patients testing negative to the standard IAA (86). Additionally, oxPTM-INS-Ab were detectable prior to clinical onset, identifying children at risk of progressing to clinical T1D (94). In the Swedish ABIS cohort, oxPTM-INS-Ab were present in 82% children progressing to clinical T1D compared to 19% in those who did not progress despite having one or more standard islet-autoantibodies after a median follow-up of 10.8 years (interquartile range 7.7-12.8). oxPTM-INS-Ab detected 17%, 26% and 44% of children in the pre-symptomatic phase who were negative to GADA, IA-2A or IAA, respectively (94). Risk for diabetes was higher (p=0.03) among children with multiple standard autoantibodies who were also oxPTM-INS-Ab^+^ compared with those who were oxPTM-INS-Ab^–^. When replacing standard insulin autoantibodies (IAA) with oxPTM-INS-Ab diabetes risk increased to 100% in children with oxPTM-INS-Ab^+^ in combination with autoantibodies to GAD (GADA) and IA-2 (IA-2A), compared to 84.37% in those with IAA/GADA/IA-2A (p=0.04) (95). Further research is ongoing to enhance assay standardization, including the evaluation of alternative techniques, and to validate these findings in larger, independent cohorts. More recently, antibodies to oxPTM-INS by •OH were investigated by electrochemiluminescence (ECL) assay and were found in 16.8% (48/258) patients with new-onset T1D. Among those, 17 patients were IAA negative confirming that oxPTM-INS-Ab may detect subjects who are negative to the gold standard IAA (96).

Mass spectrometry analyses have identified several major oxPTMs of insulin. These include chlorination of Tyr16 and Tyr26; oxidation of His5, Cys6, Cys7, Tyr16, Phe24, Phe25, and Tyr26; and glycation of Lys29 and Phe1 in the B-chain (86, 87). Notably, Tyr16, located within the B9-23 region—a known dominant epitope in autoimmune diabetes (97)—is crucial for immunoreactivity, with chlorination or oxidation potentially enhancing its immunogenicity. In a more recent analysis, additional modifications were observed in the A-chain, including oxidation of Cys6, Cys7, Cys11, Tyr14, and Cys20 (87).

These findings align with previous reports indicating that glycation predominantly affects the N-terminal Phe1 and Lys29 of the B-chain, while oxidation targets residues such as Tyr16, Tyr26, Phe24, and Cys19 (98–100). Furthermore, our group has discovered novel oxidation hotspots, including His10 and Leu17 in the B-chain, and Cys6 in the alpha-chain (87). This suggests that exposure of insulin to reactive oxidants induces diverse and widespread modifications, potentially contributing to its immunogenicity.

Exposure of insulin to reactive oxidants may also lead to oxidative cleavage. Cleavage sites resulting from oxidative damage occur preferentially between the residues phenylalanine, cysteine, glycine, leucine, valine, and tyrosine (100), as well as near the cysteine bridges, especially in chain A (99). The generation of fragments (from oxidative cleavage) may elicit immune response regardless of aminoacidic modifications. This is supported by our studies on insulin, as well as by the literature data on scleroderma where oxidatively cleaved fragments of scleroderma autoantigens are targeted by antibodies. Peptide fragments may provide neoepitopes that are ready accessible to the immune system without need for classical intracellular processing by antigen presenting cells.

More recently, our group has identified T cell specificity to oxPTM-INS peptides in people with new onset clinical T1D (87) (**Table 2**). The main response involved three insulin peptides: B:11–30, B:21–31 and A:12–21, and their respective oxPTM peptides derivatives. B:11–30 induced the strongest T cell stimulation in T1D compared with control participants for both CD4^+^ and CD8^+^ T cells. CD4^+^ response to oxPTM derivatives of the peptide B:21–31 was also commoner in T1D than in control participants (66.7% vs 27.3%). We also found antibodies to oxPTM derivatives of B:11-31 in most individuals with T1D. Of note, we observed the same pattern of response for the peptide that was oxidized in house compared with in silico-designed derivatives (ERGYYYTPKT and ERGYY-DOPA-TPKT), suggesting that oxidation of F to Y and Y to dihydroxyphenylalanine (DOPA) generates neoepitopes recognized by specific antibodies. Overall, 44.4% of patients showed a concordant autoimmune response to oxPTM-INS involving simultaneously CD4^+^ and CD8^+^ T cells and autoantibodies (87). These data are consistent with the work of Sindey et al. who have shown that peptides derived from oxPTM-INS B-chain, including the pan oxidation of ERGFFYTPKT to ERGYYYTPKT, can bind HLA class I with better affinity than unmodified insulin peptides (88) (**Table 2**). Supporting the role of oxidation, M. Kemal Aydintug et al. have presented experimental evidence that insulin peptide B:9–23-reactive γδ T cells recognize this antigen when it forms a homo-dimer due to thiol oxidation (89) (**Table 2**).

In addition to these peptide-level changes, oxidative stress has also been implicated in the induction of insulin polymers. Corichi et al. have shown the presence of insulin polymers induced by oxidation in the blood of people with obesity. Their study further revealed that the presence of the antioxidant (–)-epicatechin significantly reduces insulin polymerization, highlighting the potential of antioxidants in mitigating oxidative damage and preserving the structural integrity of insulin (101).

Oxidative PTM of insulin not only alters its immunogenic properties but might also impair its biological activity (102). For example, glycation at Phe1 in the B-chain has been shown to reduce insulin’s glucose-lowering effect, requiring 70% more glycated insulin to achieve the same effect as native insulin (98). Additionally, mixtures of glycated insulin, that may include not only mono-glycated but also di-, or tri-glycated forms (94), show a 30% reduction in its activity (103). Notably, this diminished activity appears to occur independently of insulin’s receptor binding affinity, which remains unaltered by glycation and comparable to that of the native form (98, 103).

In summary, these findings imply that immune reactivity towards oxPTM of insulin is prevalent in T1D and may precede the clinical onset by several years (94, 95). Further studies are needed to fully elucidate the mechanisms driving oxPTM generation of insulin, their variability in different pathological contexts, and their precise role in the transition from autoimmunity to beta-cell destruction.

### 4.2 GAD65

Glutamate acid decarboxylase (GAD) is an enzyme involved in the central nervous system (CNS) (104). It exists in two isoforms GAD65 and GAD67 sharing roughly 70% of sequence similarity and highly conserved C-terminal regions. However, despite these similarities, GAD65 has been identified as a major autoantigen in T1D, while GAD67 has not been specifically associated with this condition (105). GAD65 and GAD67 have been shown to have different tissue localization, with GAD65 being abundantly found within the vesicles in the pancreatic beta-cells. The localization of GAD65 within the beta-cell has made it a suggested target for oxPTMs and autoimmunity (90).

Studies have attempted to hydroxyl modify GAD65 via iron or copper sulfate catalyzation in the presence of hydrogen peroxide or ascorbic acid. The serum from T1D patients showed a predominant interaction with the highest molecular weight modified protein band of GAD. This suggests that oxidatively modified GAD interacts with antibodies in the serum of patients with T1D (91) (**Table 2**). Khan et al. demonstrated that UV radiation induction of hydroxyl modified GAD65 yielded a highly reactive autoantigen. Upon assessing the recognition of circulating antibodies in the serum of T1D patients with native or modified GAD65, they found that T1D serum was highly reactive towards ROS modified GAD65 compared to the native unmodified antigen (P<0.001) (106). Similarly, Moinuddin et al. found that ROS modified GAD65 was more immunogenic compared to native GAD65. They found that in both sera from animal models of T1D (STZ-induced diabetes in rodents) and human patients with T1D, there was a visibly stronger antibody binding towards ROS modified GAD65 compared to native GAD65 (107). Although T cell recognition of ROS-modified GAD has not been shown yet, McGinty et al. identified autoreactive T cells that were able to identify and bind to PTM modified peptides of GAD65 by citrullination and trans glutamination. The CD4^+^ T cells that were able to identify the enzymatically modified forms of GAD65 were present in higher frequencies in subjects with T1D when compared to HLA-matched controls without T1D (108).

### 4.3 Collagen Type II

Under conditions present in the inflamed joint, collagen type II (CII) undergoes modifications, leading it to function as an autoantigen in rheumatoid arthritis (RA) (109). Interestingly, RA and T1D share a notable epidemiological association, partly attributable to the overlap in genetic predisposition conferred by specific HLA alleles, such as HLA-DRB1*04, which is implicated in both diseases (92, 110, 111) (**Table 2**). Our research group showed the presence of autoantibody reactivity to oxPTM-CII also in a sub-group of patients at the clinical onset of T1D (112). In T1D, autoimmunity to oxPTM-CII showed contrasting genetic control influenced by alleles within HLA-DRB1*04 and DRB1*03. Antibody reactivity to oxPTM-CII was positively linked with DRB1*04, whereas DRB1*03 provided protective effects against native and oxPTM-CII autoimmunity in the absence of DRB1*04. When autoimmune responses to CII and oxPTM-CII were analyzed according to *HLA-DRB1* subtypes, we found that ROS-CII but not native CII autoimmunity was restricted to the HLA Shared Epitope (SE) containing DRB1*04 alleles (112), known to confer the greatest risk for developing RA (OR 3-13) (113). Besides the positive effect of the SE, we found that the HLA-DRB1*03 was negatively associated with the presence of reactivity against modified CII. The effect of each allele was also nullified when the SE and DRB1*0301 co-existed within the same individual. While the exact mechanistic link between oxPTM-CII and T1D remains unclear, shared pathways of inflammation and immune dysregulation in genetically predisposed individuals may contribute to the presentation of oxPTM-CII as an autoantigen in T1D. The ability of oxPTM-CII to interact with HLA-DRB1*04 may activate autoreactive T-cells or B-cells, as observed in RA, suggesting a possible cross-reactive mechanism in a subset of T1D patients.

It is not known yet whether oxPTM-CII reactivity can predict the development of RA (or other arthritic disorders linked to oxPTM-CII reactivity) in subjects with T1D. Our studies have demonstrated that oxPTM-CII is a highly accurate diagnostic marker for RA, with 92% sensitivity and 98% specificity, and it can even be detected in individuals with arthralgia before RA diagnosis (114). Another possibility is that oxPTM of collagen II and other types of collagens are involved in the development of chronic diabetic complications by eliciting autoimmune responses to target tissues. Some evidence suggests that autoimmune mechanisms may play a role in specific chronic complications of diabetes (115, 116). For example, Charcot neuroarthropathy (CN), a debilitating condition characterized by bone destruction and joint damage in a highly inflammatory environment (117), has been linked to increased reactivity to oxPTM-CII in patients with type 2 diabetes (118). A similar inflammatory background is seen in osteoarthritis (OA) where articular cartilage breakdown is a major characteristic. Assessment of oxPTM-CII in a mouse model of OA revealed early occurrences of oxidant production and chondrocyte hypertrophy, potentially initiating the pathological processes of OA (119).

Inflammatory arthritic disorders present overlapping symptoms, complicating diagnosis. We also evaluated oxPTM-CII autoantibodies in patients with different arthritic disorders including spondylarthritis (SpA) and RA. Our findings suggest a correlation between SpA and antibodies to oxPTM-CII. However, the antibody response differed between diseases, with IgA being predominant in SpA and IgG in RA. This difference in isotopic reactivity may point to distinct autoimmune pathways, potentially aiding in differential diagnosis (120). This evidence highlights the importance of PTM in inflammatory autoimmune conditions and their potential use as biomarkers for early diagnosis and disease stratification. Moreover, oxPTM-CII can be used to target pro-resolving biological scaffold to the arthritic joint in combination with therapeutics, suggesting the potential of this neoantigen as therapeutic target (93).

### 4.4 Prolyl-4-hydroxylase beta

Yang et al. identified carbonylated prolyl-4-hydroxylase beta (P4Hb, also known as protein disulfide isomerase A1; PDIA1) as an early autoantigen in murine models of T1D, as well as human T1D (121). P4Hb is an ER oxidoreductase, which is essential for the proper folding of insulin in pancreatic beta-cells. It functions by interacting with proinsulin to induce disulfide maturation (122). It is suggested that carbonyl modified P4Hb may be a potential target of autoimmunity and enhance the immune response towards proinsulin and insulin (**Table 2**). Notably, in NOD mouse models, autoreactive antibodies directed towards P4Hb were detected before autoantibodies towards insulin (121). Oxidative stress and influx of cytokines are two elements that have been connected to beta-cell dysfunction in diabetes and linked to amplification of carbonylation. Hence, carbonylation of P4Hb may impact and alter proinsulin processing to insulin and alter insulin folding. Yang et al. suggested that carbonylated P4Hb may be linked to the accumulation of proinsulin seen within the beta-cell of preclinical stages of T1D (123), and contribute to the following autoimmunity towards insulin as evidenced by observation that p4Hb autoantibodies may precede the onset of insulin autoantibodies (72, 121).

### 4.5 Other antigens

Few studies have assessed the immunogenicity of PTM of other two main beta-cell autoantigens, namely insulinoma antigen -2 (IA-2) and zinc transporter 8 (ZnT8). To the best of our knowledge, oxPTM of these two antigens have not been investigated yet. However, studies by Acevedo-Calado M et al. and others showed that CD4^+^ T cells may recognize *in vitro* deamidated peptides from the extracellular domain of IA-2 (IA-2ec) (124); such response is specifically targeting the 198-216, 467-482, 545-562, and 523-536 amino acid epitopes and it is elicited by endoplasmic reticulum stress through increased tissue transglutaminase (tTG) 2 activity (125). More recently, Jia et al. demonstrated that 60.6% of children with new onset diabetes were positive for autoantibodies directed towards deamidated IA-2ec compared to 19.3% positive to the wild type IA-2ec, while only 14.2% of adults with T1D and 4.1% of patients with Latent autoimmune diabetes of adults (LADA) showed autoantibodies towards deamidated IA-2 compared to 8.8% and 1.5% to the wild type IA-2ec, respectively (126). Studies have attempted to deamidate the full-length IA-2, intracellular domain of IA-2, GAD65 and ZnT8 (both ZNT8W and ZnT8R) by incubation with tTG. Donelley et al. demonstrated mixed results with the deamidation of ZnT8 and intracellular IA-2. Nonetheless, their results showed increased autoantibody binding towards tTG treated full-length IA-2 and GAD65. However, this study only included a sample size of 20 (127).

## 5 Opportunity for disease prediction and prevention

T1D prediction and prevention remain a complex and ongoing area of research. Efforts in prevention are mainly focused on identifying high-risk individuals through genetic screening and autoantibody testing, allowing early detection and intervention. Primary preventions aim to stall the onset of autoimmunity against islet autoantigens in subjects at high risk of developing T1D (128).

A secondary prevention is done for individuals displaying multiple islet autoantibodies, with the aim of stopping autoimmune processes and potentially preventing the clinical manifestation of diabetes. These strategies primarily focus on immunomodulation, aiming to halt or slow down the beta-cell destruction. Among these, teplizumab, an anti-CD3 monoclonal antibody, has emerged as a landmark intervention capable of delaying the onset of clinical T1D in high-risk individuals (129, 130). The PROTECT study and other trials have confirmed its ability to preserve beta-cell function and prolong the preclinical phase of the disease, offering hope for strategies aimed at intercepting T1D before its clinical manifestation (130). Another promising approach involves antigen-specific therapies, which seek to inactivate pathogenic autoreactive T cells in an antigen-specific manner, while preserving the overall immune function (131). Although early trials using T1D autoantigens such as insulin and GAD65 have shown limited success, recent evidence suggests potential disease-modifying effects in carefully selected patient subgroups. For instance, intralymphatic administration of GAD65 (GAD-Alum) has demonstrated significant benefits in individuals with the *HLA-DR3-DQ2* haplotype, including improved glycemic control and preservation of C-peptide levels. These findings, supported by the DIAGNODE-2 trial, highlight the importance of tailoring antigen-specific therapies to specific genetic and immunological contexts (132, 133).

Despite these advances, challenges remain. Key questions include optimal timing, dosing, and route of administration for antigen-specific therapies. It is probable that the structural format of the targeted antigen, including the possibility of PTM, may influence the efficacy of these interventions. Furthermore, to maximize the efficacy of immunotherapeutic approaches, there is an unmet need for biomarkers that bridge the gap between beta-cell health and autoimmune activation. oxPTMs might represent a promising avenue, as they may reflect both intrinsic beta-cell stress and immune activation.

The same idea can be applied to the strategies for T1D prediction which hold significant importance in averting the onset of autoimmune processes in susceptible individuals. Antibodies against PTM of beta-cell autoantigens, such as GAD65 and insulin, may play a critical role in disease onset and development. Indeed, circulating autoantibodies may detect modified forms of these antigens, potentially enhancing identification of those subjects who test negative to assay for the unmodified autoantigen (86, 134, 135). Studies are ongoing to validate the ability of these novel autoantibody specificities for early diagnosis and prediction of clinical onset of T1D (94, 96, 126). Antibodies targeting PTM and oxPTM altered proteins have been identified not only in T1D but also in various other autoimmune inflammatory conditions. There are increasing literature examples exploring the potential use of these antibodies as biomarkers. For instance, the anti-citrullinated peptide antibody assay stands as a noteworthy tool in RA diagnosis (136, 137), while deamidated gliadin peptide antibodies may help in the diagnosis of coeliac disease (138). On top of the potential predictive value, antibodies to PTM antigens may have clinical relevance to identifying sub-populations of patients such as patients more or less likely to respond to specific therapies (i.e., DMARDs, biologics). Advances in mass spectrometry, proteomics, and other analytical techniques, with the identification and characterization of PTMs, open the possibility to develop novel diagnostic tools and personalized medicine approaches that target specific modified proteins, thereby enhancing early detection and precise treatment strategies for various illnesses. The identification of PTM antigenic peptides holds promise for targeted immune tolerance development and can significantly impact diagnosis, patient characterization, and immune monitoring pre- and post-therapy. These new findings may influence the design of more effective assays for early detection and management strategies related to T1D.

## 6 Conclusions

Over the last 30 years, the role of neoepitopes in T1D has been hypothesized as a potential player in disease pathophysiology, but this topic has never been systematically studied until the last few years. Currently, advancements in biochemistry, proteomics, and the increasing availability of pancreatic islets and tissues from patients with T1D have played major roles in rapidly sparking interest in the field of autoimmunity and PTM. This progress has made it possible to unravel aspects that would have been too complicated and difficult to be studied and assessed just a decade ago. Further research is needed to fully elucidate the pathogenic role of oxPTM in beta-cell dysfunction and their potential as disease biomarkers and therapeutic targets. Understanding these processes may open new avenues for early detection and intervention, ultimately transforming our approach to T1D management and prevention.
